# Clodronate Treatment Prevents Vaginal Hypersensitivity in a Mouse Model of Vestibulodynia

**DOI:** 10.3389/fcimb.2021.784972

**Published:** 2022-01-18

**Authors:** Joel Castro, Andrea M. Harrington, Fariba Chegini, Dusan Matusica, Nick J. Spencer, Stuart M. Brierley, Rainer V. Haberberger, Christine M. Barry

**Affiliations:** ^1^ Visceral Pain Research Group, College of Medicine and Public Health, Flinders Health and Medical Research Institute (FHMRI), Flinders University, Bedford Park, SA, Australia; ^2^ Hopwood Centre for Neurobiology, Lifelong Health Theme, South Australian Health and Medical Research Institute (SAHMRI), Adelaide, SA, Australia; ^3^ Musculoskeletal Neurobiology Laboratory, College of Medicine and Public Health, Flinders Health and Medical Research Institute (FHMRI), Flinders University, Adelaide, SA, Australia; ^4^ Pain and Sensory Neurobiology Laboratory, College of Medicine and Public Health, Flinders Health and Medical Research Institute (FHMRI), Flinders University, Adelaide, SA, Australia; ^5^ Visceral Neurophysiology Laboratory, College of Medicine and Public Health, Flinders Health and Medical Research Institute (FHMRI), Flinders University, Adelaide, SA, Australia; ^6^ School of Biomedicine, Faculty of Health and Medical Sciences, University of Adelaide, Adelaide, SA, Australia

**Keywords:** vulvodynia, vestibulodynia, macrophage, visceromotor reflex, nociception, clodronate peripheral nociceptor

## Abstract

**Introduction:**

Improved understanding of vestibulodynia pathophysiology is required to develop appropriately targeted treatments. Established features include vulvovaginal hyperinnervation, increased nociceptive signalling and hypersensitivity. Emerging evidence indicates macrophage-neuron signalling contributes to chronic pain pathophysiology. Macrophages are broadly classified as M1 or M2, demonstrating pro-nociceptive or anti-nociceptive effects respectively. This study investigates the impact of clodronate liposomes, a macrophage depleting agent, on nociceptive signalling in a mouse model of vestibulodynia.

**Methods:**

Microinjection of complete Freund’s adjuvant (CFA) at the vaginal introitus induced mild chronic inflammation in C57Bl/6J mice. A subgroup was treated with the macrophage depleting agent clodronate. Control mice received saline. After 7 days, immunolabelling for PGP9.5, F4/80+CD11c+ and F4/80+CD206+ was used to compare innervation density and presence of M1 and M2 macrophages respectively in experimental groups. Nociceptive signalling evoked by vaginal distension was assessed using immunolabelling for phosphorylated MAP extracellular signal-related kinase (pERK) in spinal cord sections. Hyperalgesia was assessed by visceromotor response to graded vaginal distension.

**Results:**

CFA led to increased vaginal innervation (p < 0.05), increased pERK-immunoreactive spinal cord dorsal horn neurons evoked by vaginal-distension (p < 0.01) and enhanced visceromotor responses compared control mice (p < 0.01). Clodronate did not reduce vaginal hyperinnervation but significantly reduced the abundance of M1 and M2 vaginal macrophages and restored vaginal nociceptive signalling and vaginal sensitivity to that of healthy control animals.

**Conclusions:**

We have developed a robust mouse model of vestibulodynia that demonstrates vaginal hyperinnervation, enhanced nociceptive signalling, hyperalgesia and allodynia. Macrophages contribute to hypersensitivity in this model. Macrophage-sensory neuron signalling pathways may present useful pathophysiological targets.

## 1 Introduction

Localised provoked vestibulodynia is the most common form of clinical vulvodynia and involves pain at the vaginal entrance provoked by local touch or vaginal distention (Akopians and Rapkin, 2015). Key pathophysiological features include hyperinnervation, hypersensitivity and increased abundance of immune cells (Bohm-Starke et al., 1999; Bohm-Starke et al., 2001; Bornstein et al., 2004). Many therapies have failed to show benefit in clinical trials, and none have directly targeted vulvodynia pathophysiology (Nunns et al., 2010; Stockdale and Lawson, 2014; Falsetta et al., 2017).

To investigate vestibulodynia pathophysiology we developed a mouse model of hyperinnervation in response to mild chronic inflammation, induced by microinjection of complete Freund’s adjuvant (CFA) at the vaginal entrance (Sharma et al., 2018). Normal innervation of the vagina includes multiple populations of nerve fibres, most abundant in the lamina propria (Barry et al., 2017) and hyperinnervation in our model involves multiple populations of neurons, consistent with clinical vestibulodynia (Bohm-Starke et al., 1999). Increased CD68+ putative macrophages accompanies vaginal hyperinnervation in our model of vestibuldynia (Sharma et al., 2018). However, the role of macrophages in this model, and in clinical vestibuldynia, is unclear.

Recent studies highlight macrophage-neuron signalling that promotes axonal sprouting and nociceptor sensitization, potentially significant in vulvodynia pathophysiology (Wernli et al., 2009; Shepherd et al., 2018; Barry et al., 2019; Kolter et al., 2019; Warwick et al., 2019). Macrophages are heterogenous, highly plastic and capable of releasing hundreds of effector molecules depending on their functional state (Gordon and Taylor, 2005; Cassetta et al., 2011; Lawrence and Natoli, 2011). Their phenotype alters rapidly in response to changes in the microenvironment (Gordon et al., 2014), and they can contribute to the induction and the resolution of inflammation.

A simplified and robust framework classifies macrophages as M1, with proinflammatory and pro-nociceptive effects and M2, with anti-inflammatory and anti-nociceptive effects (Mantovani et al., 2005; Martinez and Gordon, 2014; Viola et al., 2019). Within these classes, different macrophage phenotypes express distinct combinations of cell surface receptors. CD68 and F4/80 are widely used as pan-macrophage markers. Combined immunoreactivities for F4/80 together with CD11c or CD206 have been used to histologically identify putative M1 and M2 macrophages respectively (Leung et al., 2016; Zhu et al., 2017; James et al., 2018).

Vaginal hypersensitivity accompanies hyperinnervation in rodent models of vestibuldynia induced by repeated fungal infection (Farmer et al., 2011; Hoheisel et al., 2015) or injection of CFA (Chakrabarty et al., 2018). In both studies mechanical sensitivity was assessed using graded monofilaments. A quantitative measure of changes to nociceptive signalling is MAP extracellular signal-related kinase (ERK) in dorsal horn neurons where it is rapidly phosphorylated (pERK) by noxious, but not non-noxious stimuli (Ji et al., 1999). ERK activation increases neuronal sensitivity to further inputs, mediating central sensitisation (Ji et al., 1999; Gao and Ji, 2009). Another highly reproducible and reliable quantitative measure of visceral nociception is the visceromotor response (VMR) (Ness and Gebhart, 1988), a nociceptive brainstem reflex in which noxious distension of hollow organs such as the vagina triggers contraction of abdominal muscles (Berkley et al., 2007; Ge et al., 2019; Castro et al., 2021). Assessing pERK and VMR in our model will show whether the nociceptive signal sent from the periphery to the CNS is augmented, and whether this ultimately results in vaginal hypersensitivity associated with chronic pain.

Therefore, this study has three distinct aims. First, to identify the subtypes of macrophages that accompany hyperinnervation in our model and next to identify if our model is associated with increased nociceptive sensitivity. Finally, by assessing the impact of macrophage depletion, we aim to identify if macrophages contribute to vaginal hyperinnervation, enhanced nociceptive signalling and vaginal hypersensitivity in this model of vestibulodynia.

## 2 Materials and Methods

### 2.1 Ethical Approval

All experiments were performed according to relevant regulatory standards, ARRIVE guidelines, and guidelines established by the National Health and Medical Research Council of Australia. The Animal Welfare Committee of Flinders University approved all experiments involving animals (AEM1582).

### 2.2 Induction of Inflammation

In female mice (C57Bl/6J, 6 to 8 weeks of age, independent of the oestrus cycle) mild chronic inflammation was induced in the vagina according to procedures previously described (Sharma et al., 2018). Under general anaesthesia (isoflurane, induction 4%, maintenance 1.5% in oxygen), CFA (5 µl, Sigma-Aldrich, Missouri, USA) was injected into the vaginal wall at the introitus using a pulled glass micropipette. Control mice received an equivalent volume of 0.9% saline injection. Following intravaginal injections anaesthesia was withdrawn and mice recovered. Mice were monitored at least twice daily for 7 days for evidence of pain or dysfunction.

### 2.3 Depletion of Vaginal Macrophages

A subgroup of mice that received CFA was treated with clodronate (ca. 5 mg/ml) to deplete macrophages in the vagina. Clodronate liposomes (Liposoma Technology, Netherlands) were administered on day 0 (180 µl I.P., 10 µl intravaginal) and day 4 (180 µl I.P.). Control mice received the same procedures with an equivalent volume of saline injected.

### 2.4 Dorsal Horn Neuron Activation Evoked by *In Vivo* Vaginal Distension

Following VMR measurements mice underwent acute noxious vaginal distension immediately prior to transcardial perfusion fixation. After which the lumbosacral spinal cord was removed and processed for immuno-labelling of the neuronal activation marker phosphorylated extracellular signal-related kinase (pERK) to compare the extent of spinal cord dorsal horn neuronal activation between experimental groups. After the completion of VMR measurements, the balloon catheter was removed from the vaginal canal and mice were returned to home cage. After 90 min, mice were anaesthetised, during which the balloon catheter re-inserted into the vaginal canal and secured to the tail. Mice were removed from isoflurane chamber and as they regained consciousness the balloon catheter was distended to 60 mmHg (5 x 20 s distension with 5 s deflation interval) *via* a syringe attached to a sphygmomanometer pressure gauge. After the final distension, mice were given an overdose of euthanasia agent (intraperitoneal pentobarbitone, Lethabarb, Virbac, Australia) and within 5 min undergone transcardial perfusion fixation with 4% paraformaldehyde in 0.1 M phosphate buffer.

### 2.5 Tissue Fixation, Processing and Sectioning

After pentobarbitone overdose, the chest cavity was opened, and 0.5 mL of heparin saline was injected into the left ventricle. The right atrium was then snipped, allowing for perfusate drainage. Warm 0.1M phosphate buffer was perfused into the left ventricle followed by ice-cold 4% paraformaldehyde in 0.1 M phosphate buffer (Sigma-Aldrich, St. Louis, MO).

After complete perfusion, the lumbosacral spinal cord was removed from below vertebrae L1 through to vertebrae L6 and post-fixed in 4% paraformaldehyde in 0.1M phosphate buffer at 4°C for 18 to 20 hours. Spinal cord was transferred to 30% sucrose in 0.1M phosphate buffer then into 50% OCT/30% sucrose phosphate buffer prior to freezing in 100% OCT using liquid nitrogen cooled isopentane. Frozen spinal cord samples were sectioned on a cryostat (Leica CM 1950) with 10 µm thick cross-sections of L6-S1 spinal cord placed onto slides (InstrumeC Uberfrost Printer Slides) and stored at -20°C prior to pERK immuno-labelling. Sections of L6-S1 spinal cord levels were recognised using the Allen Spinal Cord Atlas (Allen Institute for Brain Science, 2008).

The vagina together with the urethra was isolated and immersed overnight at 4°C in Zamboni’s fixative (2% formaldehyde; 0.5% picric acid; 0.2M sodium phosphate buffer, pH 7.0), then dehydrated in ethanol (80% 3 x 20 min, 90% 1 x 30 min, 100% 2 x 30 min), cleared in xylene (2 x 30 minutes) and rehydrated through a series of ethanol washes (100% 2 x 30 min, 80% 1 x 30 min, 50% 1 x 30 min) to water (1 x 30 min). Tissue was transferred into 30% sucrose in phosphate buffered saline (PBS) overnight at 4°C then shock frozen in OCT compound (Tissue-Tek, Miles, USA). Cryostat sections were cut at 10 µm thickness, mounted on polyethyleneimine-coated slides and vacuum dried for 30 minutes. For each histological stain and each combination of antibodies, four sections from the vagina per mouse were labelled, with at least 100 µm distance between sections labelled with the same stain or antibody combination.

### 2.6 Histochemistry

Phosphorylated MAP kinase ERK 1/2 (pERK) immuno-labelling was performed on spinal cord sections from the different experimental groups mixed together in numerous batches using a DAKO Omnis auto-stainer. The primary antibody (pERK 1/2, 1:800, MAB4370, Cell Signalling Technology, Genesearch, Qld) diluted in antibody Diluent (S0809, Agilent DAKO, Santa Clara, CA) was detected with 3,3′-Diaminobenzidine (DAB)/horseradish peroxidase (HRP) secondary antibody staining. Non-specific binding of secondary antibodies was blocked with Serum-Free Protein Block (X0909, Agilent DAKO). Tissue sections were pre-incubated with primary antisera for 1 hour, washed and incubated in Envision FLEX Peroxidase-blocking Reagent (GV823, Agilent DAKO), followed by Envision FLEX HRP Polymer (GV823, Agilent DAKO) for HRP binding. Sections were then washed in wash buffer (GC807, DAKO Omnis, Agilent) before a 10-minute incubation in EnVision FLEX Substrate Working Solution (DAB).

Haematoxylin and eosin (H&E) staining of vaginal sections was used to visualise morphology and assess swelling (oedema) associated with inflammation. Sections were washed in PBS and stained according to standard protocols (haematoxylin 6 min, 1% acid alcohol 5 s, lithium carbonate 4 min, eosin 2 min, 100% ethanol 3 x 10 s and xylene 2 x 2 min). Sections were washed under running water (1 min) after immersion in each reagent before dehydration in ethanol. Slides were coverslipped using DePex mounting medium (BDH Chemicals, Poole, UK).

Multiple labelling immunohistochemistry of vaginal sections was used to identify nerve fibres immunoreactive to PGP9.5 and cells immunoreactive for F4/80 (as a pan-macrophage marker), CD11c (as a marker of classically activated macrophages) and CD206 (as a marker of alternatively activated macrophages) (Kigerl et al., 2009). The F4/80 monoclonal antibody developed in Gordon’s laboratory is widely used as a macrophage-specific marker, and labels both resident and blood-derived macrophages (Austyn and Gordon, 1981). CD11c is a type 1 transmembrane protein expressed by proinflammatory, M1-like macrophages (Lumeng et al., 2008). CD206, also known as mannose receptor, is a membrane protein and pattern recognition receptor widely used as a molecular signature of M2-like macrophage activation state (Viola et al., 2019).

Non-specific binding was blocked using 10% normal donkey serum in PBS (60 minutes), and sections were incubated in combinations of primary antisera (**Table 1**) for 48 hours in a humid chamber at room temperature. Sections were washed in PBS (3 x 10 min), incubated in combinations of secondary antisera (**Table 1**) for 2 hours at room temperature, washed in PBS (3 x 10 min) and coverslipped in carbonate-buffered glycerol at pH 8.6.

**Table 1:** Antibodies and antisera used to label vaginal sections.

Antigen	Dilution	Host	Supplier
PGP9.5	1 to 1000	Rabbit	Cedarlane
CD206 (biotin conjugated)	1 to 50	Rat	BioLegend
F4/80	1 to 1000	Rat	BioRad
CD11c	1 to 200	Armenian Hamster	BioLegend
**Secondary antisera and streptavidin conjugates**			
FITC Donkey anti Rabbit	1 to 100	Jackson ImmunoResaerch	
CY3 Donkey anti Rat	1 to 100	Jackson ImmunoResaerch	
CY3 Streptavidin	1 to 100	Jackson ImmunoResaerch	
CY5 Donkey anti Hamster	1 to 50	Jackson ImmunoResaerch	
CY5 Donkey anti Rat	1 to 50	Jackson ImmunoResaerch	

### 2.7 Microscopy and Image Analysis

Immunolabelled spinal cord sections were imaged using a NanoZoomer slide scanner (Hamamatsu, Japan) with a 40x objective. At the time of scanning, the images produced were assigned a random number that de-identifies their experimental group. The scanned images were then opened and viewed using free NDPview2 software (https://www.hamamatsu.com/jp/en/product/type/U12388-01/index.html). The images were not manipulated in any way.

Neuronal counts in the dorsal horn of spinal cord sections L6-S1 were performed using the scanned images opened in digital pathology viewing software QuPath 0.1.2. The mean number of pERK-immunoreactive (IR) dorsal horn neurons/section was obtained from each mouse from 5-10 sections. The mean number +/- SEM of pERK-IR/section was compared between experimental groups and statistically compared using parametric or non-parametric one-way ANOVA with appropriate *post-hoc* comparison tests (Prism 9 analysis software) with normality Shapiro-Wilk test used to assess the normal distribution of data.

Sections stained for H&E were viewed using brightfield microscopy (Olympus BX53) and images acquired using a 4 x objective lens. To assess oedema, the cross-sectional area of the lamina propria was measured using the freehand selection tool and measure function in ImageJ.

Immuno-labelled vaginal sections were imaged using an epifluorescence microscope (Olympus BX50, Tokyo, Japan) with selective excitation LED (CoolLED Ltd, Hampshire, UK) and emission filter combinations. Images were acquired using a 40 x objective lens, CoolSNAP fx monochromatic camera (Photometrics, Az, USA) and MicroManager Imaging Software (UCSF, Ca, USA). Images were imported to ImageJ (NIH, Bethesda, MD, USA) and over image stacks created. Immunolabelled cells (F4/80+CD11c+ and F4/80+CD206+) were manually quantified in 12 regions of interest per mouse (3 images per section, 4 sections per mouse) by a researcher blinded to the animal’s treatment group. For quantification of nerve fibres, a 9 x 7-point grid was overlaid using ImageJ’s digital Grid tool and immunoreactive nerve fibres intersecting with the grid were quantified.

### 2.8 *In Vivo* Visceromotor Response to Vaginal Distension

On day 7 we recorded the visceromotor response (VMR) to vaginal distension (VD) as an objective measurement of vaginal sensitivity to pain in fully conscious animals (Berkley et al., 2007; Ge et al., 2019; Castro et al., 2021). Abdominal muscle contraction in response to non-noxious and noxious VD was quantified by recording the electrical activity (EMG) as previously described (Castro et al., 2021).

#### 2.8.1 Implantation of Electromyography (EMG) Electrodes

Under isoflurane anaesthesia, the bare endings of two Teflon-coated stainless-steel wires (Advent Research Materials Ltd, Oxford, UK) were sutured into the right abdominal muscle and tunnelled subcutaneously to be exteriorized at the base of the neck for future access. At the end of the surgery, mice received prophylactic antibiotic (Baytril^®^; 5mg/kg s.c.) and analgesic (buprenorphine; 0.05 mg/kg s.c.) and were housed individually and allowed to recover for at least three days before assessment of VMR.

#### 2.8.2 Assessment of Visceromotor Responses

VMR was assessed in all three groups of mice: control (saline), CFA and CFA plus clodronate. On day 7 mice were briefly anaesthetized using inhaled isoflurane and a lubricated latex balloon of ~3 mm length was gently introduced through the vagina and inserted up to 1 mm proximal to the vaginal verge. The balloon catheter was secured to the base of the tail and connected to a barostat (Isobar 3, G&J Electronics, Willowdale, Canada) for graded and pressure-controlled balloon distension. Mice were allowed to recover from anaesthesia in a restrainer with dorsal access for 10 minutes prior to initiation of the distension sequence. Graded distensions were applied at 20-30-40-60-80 mmHg for 30 s duration each, at 4 min intervals. The EMG electrodes were relayed to a data acquisition system and the signal was recorded (NL100AK headstage), amplified (NL104), filtered (NL 125/126, Neurolog, Digitimer Ltd, bandpass 50–5000 Hz) and digitized (CED 1401, Cambridge Electronic Design, Cambridge, UK) to a PC for off-line analysis using Spike2 (Cambridge Electronic Design).

### 2.9 Statistical Analysis

For VMR data, the analogue EMG signal was rectified and integrated. To quantify the magnitude of the VMR at each distension pressure, the area under the curve (AUC) during the distension (30 s) was corrected for the baseline activity (AUC pre-distension, 30 s). Total area under the cure (Total AUC) was quantified by adding the individual AUC at each distension pressure. VMR data were statistically analysed by generalised estimating equations followed by LSD *post hoc* test when appropriate using SPSS 27.0.

Frequency data were analysed using analysis of variance (ANOVA) followed by appropriate post-tests in GraphPad Prism 9 Software, San Diego, CA, USA, with normality Shapiro-Wilk test used to assess the normal distribution of data. Figures were prepared in GraphPad Prism and data are presented as mean ± standard error of the mean (SEM). Asterisks indicate where post-tests show differences. N represents the number of animals and a *p* value < 0.05 is considered significant.

## 3 Results

### 3.1 Overview

CFA was associated with vaginal hyperinnervation, increased ERK activation in dorsal horn neurons and increased VMR. The increased number of pERK-IR dorsal horn neurons and the elevated VMR were prevented by administration of clodronate. In mice that received CFA, clodronate administration was also associated with fewer putative M1 and M2 macrophages in the vagina.

### 3.2 Clodronate Treatment Reduced M1 and M2 Macrophages in Mice That Received CFA

**Figure 1:**
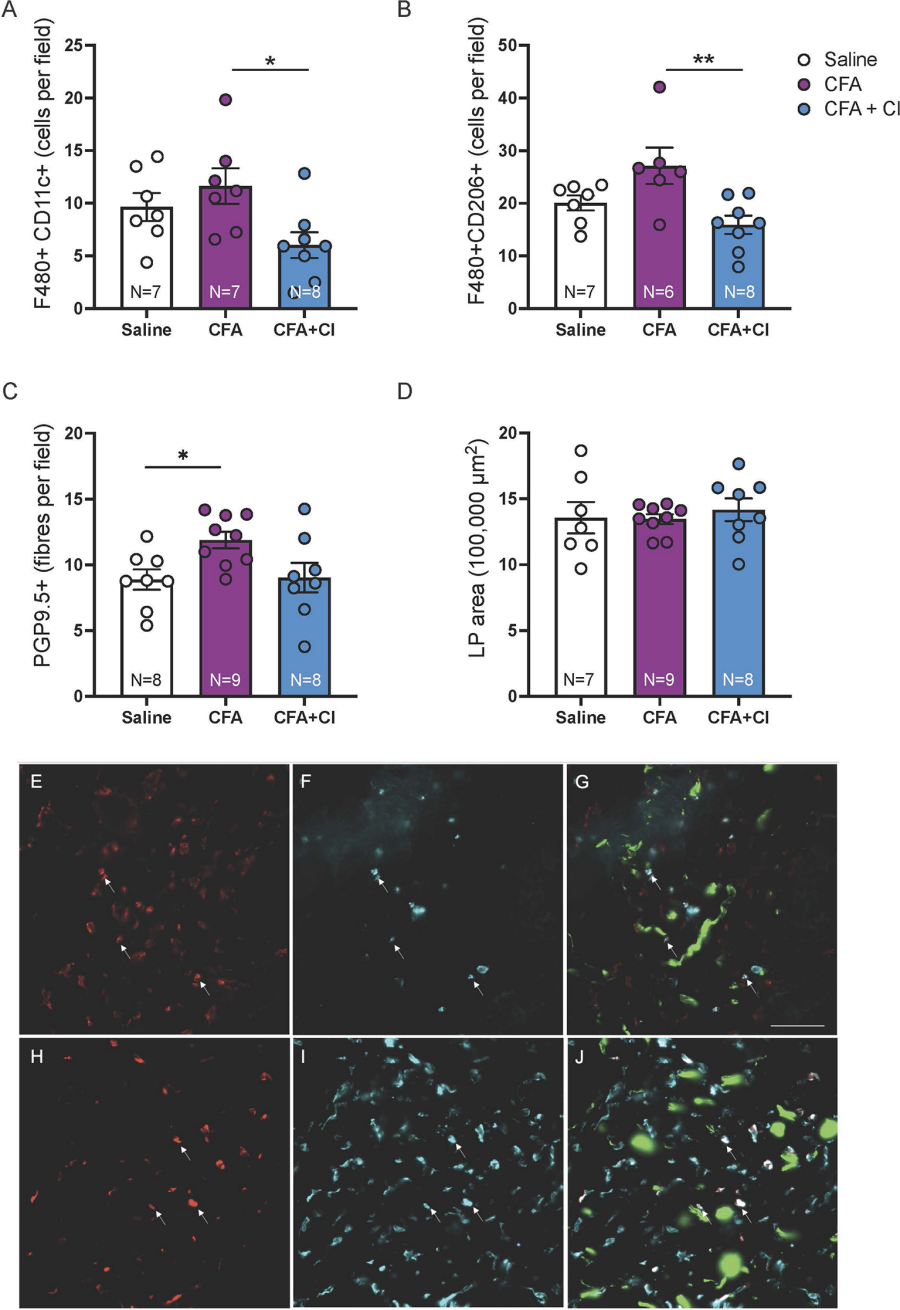
Clodronate treatment depleted vaginal macrophages in the CFA mouse model of vulvodynia. **(A)** Putative M1 macrophages were identified less frequently in F480/CD11c-immunolabelled vaginal sections from mice that received CFA and were treated with clodronate (*p < 0.05, ANOVA: F_(2,19)_ = 4.20, p = 0.03, control saline 9.6 ± 1.3; CFA 11.6 ± 1.6; CFA + clodronate 6.0 ± 1.2). **(B)** Similarly, putative M2 macrophages, identified by immunolabelling for F4/80 and CD206, were found less frequently in sections from mice that were treated with clodronate (**p < 0.01, ANOVA: F_(2,18)_ = 6.37, p = 0.008, control saline 20.1 ± 1.4; CFA 27.1 ± 3.5; CFA + clodronate 15.9 ± 1.7). **(C)** The number of nerve fibres immunoreactive for the pan-neuronal marker PGP9.5 was higher in mice that received CFA compared to control mice (*p < 0.05) but not significantly reduced in mice treated with clodronate (ANOVA: F_(2,22)_ = 4.10, p = 0.03; control saline 8.9 ± 0.8; CFA 11.9 ± 0.6; clodronate: 9.0 ± 1.1, N = 8 per group). **(D)** Comparison of vaginal cross-sectional lamina propria (LP) area indicated no evidence of swelling (oedema) in any group one week following administration of CFA with or without clodronate (ANOVA: F_(2,23)_ = 0.045, p = 0.96, N = 7 – 9 per group). **(E–G)** Example images of putative M1 macrophages labelled with F4/80 **(E)** and CD11c **(F)** and overlay image **(G)** showing F4/80+CD11c+ cells (arrows) plus nerve fibres (green) immunoreactive for PGP9.5. **(H–J)** Example images of putative M2 macrophages labelled with F4/80 **(H)** and CD206 **(I)** and overlay image **(J)** showing F4/80+CD206+ cells (arrows) and PGP9.5-IR nerve fibres (green). These images are from a mouse that received CFA. Scale bar = 50 µm.

To compare the abundance of putative M1 and M2 macrophages in groups of mice, macrophages co-labelled for the pan macrophage marker F4/80 and the M1 marker CD11c or the M2 marker CD206 were quantified in vaginal sections (4 sections, 12 ROI per mouse). Mice that received CFA display increased abundance of putative M1 macrophages (F4/80+ CD11c+, p < 0.05) and M2 macrophages (F4/80+CD206+, p < 0.01), compared to mice treated with clodronate (**Figures 1A, B**). We found that treatment with clodronate reduces both putative M1 macrophages (p < 0.05, CFA 11.6 ± 1.6; CFA + clodronate 6.0 ± 1.2, **Figures 1A**, **E**–**G**), and putative M2 macrophages (p < 0.01, CFA 27.1 ± 3.5; CFA + clodronate 15.9 ± 1.7, **Figures 1B**, **H**–**J**).

### 3.3 Vaginal Hyperinnervation Was Induced by CFA and Not Prevented by Clodronate

To assess the impact of clodronate treatment on hyperinnervation induced by CFA, PGP9.5+ nerve fibres were quantified in vaginal sections (4 sections, 12 ROI per mouse). Vaginal innervation (mean PGP9.5+ fibres per field) is increased 7 days after administration of CFA (p < 0.05) and no change associated with clodronate treatment was identified (control 8.9 ± 0.8; CFA 11.9 ± 0.6; clodronate: 9.0 ± 1.1, **Figure 1C**). No evidence of vaginal swelling (increased lamina propria (LP) area) was identified in any group (p > 0.05, **Figure 1D**).

### 3.4 Spinal Cord Neuron Activation Was Increased by CFA and Sensitisation Was Prevented by Clodronate

**Figure 2:**
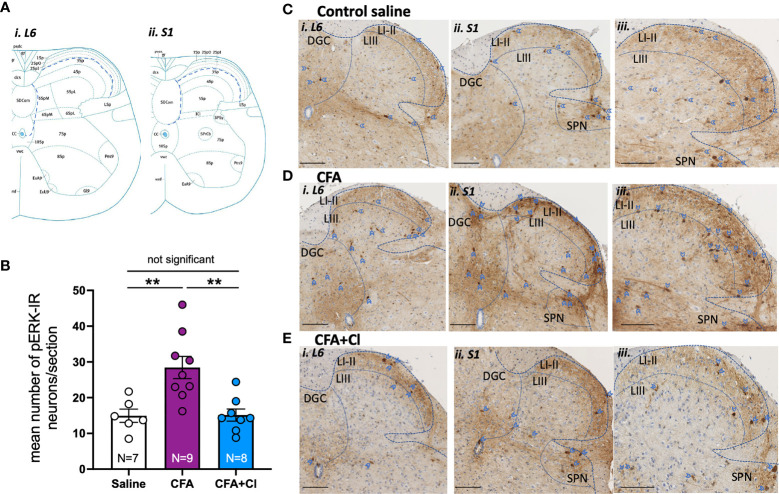
Clodronate liposome treatment revert the CFA-induced increase in the nociceptive signalling sent to the central nervous system. **(A)** Schematic demonstration of dorsal horn laminae in spinal cord in cross section, demonstrating areas in which pERK-IR neurons evoked by vaginal distension were quantified within *i)* L6 and *ii)* S1 (Allen Institute for Brain Science, 2008). **(B)** The number of pERK immunoreactivity (pERK-IR) evoked by *in vivo* vaginal distension (60 mmHg) was quantified within the lumbosacral L6 and S1 spinal cord in control saline mice (clear bar), mice that had received complete Freud’s adjuvant (CFA; plum bar) and mice that received CFA and were treated with clodronate (CFA+Cl; blue bar). Grouped data showed a significant increase (**p < 0.01) in the number of pERK-IR neurons/section across the L6-S1 dorsal horn of the spinal cord in mice that received CFA compared to control saline mice. This increase in dorsal horn neuron activation was not observed in CFA mice treated with clodronate, with the number of pERK-IR neurons significantly (**p < 0.01) reduced in CFA-clodronate mice relative to CFA-treatment mice. ANOVA: F_(2,20)_ = 10.20, p = 0.0009, control saline 15.0 ± 1.9; CFA 28.4 ± 3.1; CFA + clodronate 15.1 ± 1.7, data represents mean ± SEM pERK-IR neurons per section of LS spinal cord, from 5-10 sections per mouse. **(C–E)** Representative images of pERK-IR neurons (dark brown, blue arrow heads) in cross sections of *i)* L6 and ii and *iii)* S1 spinal cord dorsal horn from **(C)** Mice that received saline, **(D)** mice that received CFA and **(E)** CFA mice that received intravaginal and intraperitoneal injections of clodronate. Scale bars = 100 µm. pERK-IR neurons were observed in superficial dorsal horn laminae I and II (LI-II) and in lamina III (LIII), as well as in the dorsal grey commissure (DGC) and in the sacral parasympathetic nuclei (SPN) in sacral sections.

We then assessed whether CFA-induced alteration in vaginal pain sensitivity is driven by an increase in the nociceptive signal sent from the vagina to the central nervous; and whether clodronate-induced macrophages depletion could revert this aberrant pain signal. For this, we quantified the activation of extracellular signal-related kinase (ERK) within spinal cord dorsal horn neurons, evoked by noxious (60mmHg) vaginal distension. The number of pERK-immunoreactive (IR) neurons (activated ERK) evoked by acute noxious vaginal distension was quantified in the dorsal horn of the lumbosacral (L6-S1) spinal cord levels (**Figure 2A**). In control (saline) mice, noxious distension of the vagina (60mmHg) induces activation of ERK neurons within the superficial dorsal horn laminae I-III, in the dorsal grey commissure (DGC), and in the sacral parasympathetic nuclei (SPN) (**Figures 2B, C**). pERK-IR neurons are similarly distributed in the dorsal horn of CFA mice treated without (**Figure 2D**) and with clodronate (**Figure 2E**).

However, there are significantly more (p <0.01) pERK-IR neurons in mice that received CFA (28.4 ± 3.1 neurons/section; **Figure 2B, D**) relative to control saline (15.0 ± 1.9 neurons/section; **Figure 2B, C**). Clodronate liposome injections significantly reduce CFA-induced elevation of pERK-IR neurons within the LS region of the spinal cord, normalising ERK activation to that observed in healthy control (saline) mice *(*15.1 ± 1.7 neurons/section, **Figure 2B, E**).

### 3.5 Visceromotor Response Is Increased in Mice That Received CFA and Reduced by Clodronate

**Figure 3:**
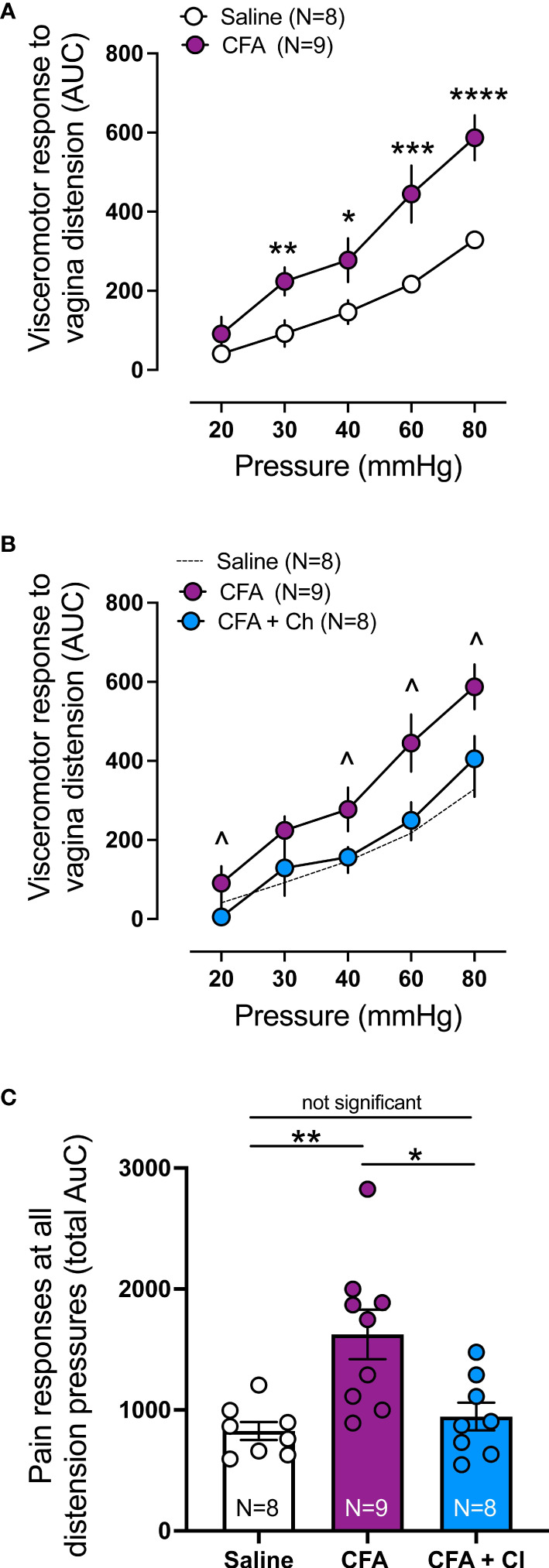
Clodronate treatment decreases the *in vivo* enhanced nociceptive responses to vaginal distension in a model mice of vulvodynia. **(A)** Visceromotor response (VMR) to vagina distension (VD) was significantly increased in animals treated with Complete Freud Adjuvant (CFA), compared to control (saline) treated animals (**p ≤ 0.01 at 30mmHg; *p ≤ 0.05 at 40mmHg, ***p ≤ 0.001 at 60, and ****p ≤ 0.0001 at 80mmHg, generalised estimating equations followed by LSD *post hoc* test, from N=8-9 mice). Data expressed as area under the curve of the corresponding EMG signal. Data represent Mean ± SEM. **(B)** Treatment with clodronate liposomes in mice that received CFA (CFA + Cl) significantly reduced the elevated VMR to vagina distension present in mice that received CFA alone (CFA), normalising the responses to control mice (saline, N=8). (^ p ≤ 0.05 at 20, 40, 60 and 80 mmHg, generalised estimating equations followed by LSD *post hoc* test, from N=8-9 mice). Data expressed as area under the curve of the corresponding EMG signal. Data represent Mean ± SEM. CFA and Saline groups are the same as presented in **(A)**. **(C)** Grouped data expressed as the total area under the curve (AUC) of the VMR to VD showing significantly increased responses in CFA treated mice compared with control saline mice. Treatment with clodronate liposomes (Cl) reversed the increased responses present in CFA animals back to control saline levels (Ordinary one-way ANOVA followed by Bonferroni *post hoc* test, from N=8-9 mice). Each dot represents the total AUC from an individual animal. Data represent Mean ± SEM.

Visceromotor response (VMR) to vaginal distension (VD) was assessed to identify if CFA induced vaginal hypersensitivity, and whether clodronate depletion could restore normal vaginal sensitivity, hence reduce chronic pain associated with vestibulodynia. Compared to control (saline) mice, VMR is significantly increased in mice that received CFA at 30mmHg (p < 0.01), 40mmHg (p < 0.05), 60 mmHg (p < 0.001) and 80 mmHg (p < 0.0001) (**Figure 3A**). Treatment with clodronate liposomes reduces increased VMR to VD observed in CFA treated mice, normalizing vagina sensitivity to that of control (saline) animals (**Figures 3B, C**).

## 4 Discussion

This study demonstrates for the first time increased spinal cord neuron activation and increased VMR associated with vaginal hyperinnervation in a model of vestibulodynia. Clodronate liposomes deplete macrophages of multiple phenotypes and prevents vaginal hypersensitivity, indicating signalling involving macrophages or other signalling pathways play a significant role in this model of vulvodynia.

Our model replicates two major features of clinical vestibulodynia: hyperinnervation and mechanical allodynia at the vaginal entrance. Established models of provoked vestibulodynia include a mouse model induced by repeated candida albicans exposure (Farmer et al., 2011) and a rat model induced by injection of CFA in vestibular tissue adjacent the vaginal entrance (Chakrabarty et al., 2018). In these models and ours, vulvovaginal hyperinnervation is demonstrated by immunohistochemical labelling of nerve fibres in sections of vulvovaginal tissue. The vulva of mice is anatomically different to humans with no labia majora or minora (Cunha et al., 2020), and the average length of the mouse vagina is 4.96 mm (Robison et al., 2017), compared to 62.7 mm from introitus to cervix in women (Barnhart et al., 2006). In previously established models, mechanical hypersensitivity has been demonstrated by responses to stimuli with graded monofilaments to the vulvovaginal area. In our study, allodynia to distension was demonstrated using a balloon inserted only 1 mm beyond the vaginal entrance. This result has correlation with pain on vaginal penetration, a hallmark symptom of clinical vulvodynia.

In mice that received CFA, clodronate treatment reduced putative M1 (F480+CD11c+) and M2 (F4/80+CD206+) macrophages by 50% and 40% respectively. The population of vaginal macrophages include resident macrophages that self-renew *in situ* and whose progenitors originate embryonically in yolk sac and foetal liver, and macrophages of bone marrow origin that terminally differentiate in the vagina from circulating monocytes after migrating in response to inflammation (Hoeffel et al., 2012; Hashimoto et al., 2013). Clodronate administered *via* liposomes enters macrophages *via* phagocytosis leading to apoptotic cell death (Selander et al., 1996; Van Rooijen et al., 1996). Given the capacity for macrophage replacement from resident and circulating cells, macrophage depletion following clodronate administration is transient and in parallel with depletion, inflammatory cytokines recruit circulating monocytes which migrate and differentiate to replace macrophages in tissue. Therefore, the observed partial reduction of macrophages following 2 administrations of clodronate over 7 days is expected.

This partial depletion of macrophages is associated with substantial protection against vaginal hypersensitivity, as demonstrated by reduced VMR to vaginal distension (VD) *in vivo*. Further studies are required to identify the mechanisms by which clodronate ameliorates allodynia and hyperalgesia in response to CFA in this model. Multiple receptors and signalling molecules may contribute including activation of toll-like receptor 4 (TLR4) in macrophages which can induce synthesis and release of proinflammatory chemokines and cytokines including IL1b, IL6 and TNF-a (Swanson et al., 2020). Other models and clinical studies indicate lymphocytes may also contribute to vestibulodynia (Akopians and Rapkin, 2015; Tommola et al., 2015; Liao et al., 2017). Elucidating the mechanisms involved will help identify useful therapeutic targets. Clodronate has demonstrated benefit in clinical trials for treatment of bone and joint pain, with analgesic effects independent of inhibition of bone resorption (Kim et al., 2013; Rossini et al., 2015; Saviola et al., 2017). Further studies are required to demonstrate the extent to which these beneficial effects may occur *via* mechanisms independent of macrophages. Our results suggest further studies to identify mechanisms by which clodronate protects against vaginal hypersensitivity in this model are warranted.

An increase in the nociceptive signal sent to the central nervous system is evident in mice that received CFA. This is demonstrated by the increased pERK activation evoked by noxious VD in dorsal horn neurons located in the lumbosacral spinal cord (L6-S1), which are the very same segments that received sensory input from the vagina (Inyama et al., 1986; Ge et al., 2019). As with VMR, the pERK data clearly distinguishes control mice compared those that received CFA, which contain almost double the number of activated neurons. Supporting findings from VMR data, the pERK data shows enhanced neuronal activation in response to CFA is prevented by administration of clodronate, possibly mediated by and the substantial depletion of macrophages.

This is the first time that vulvovaginal sensitivity has been assessed *in vivo* using VMR to VD in a model of vaginal hyperinnervation, and it clearly discriminates CFA mice from control as well as clodronate-treated mice. Increased VMR elicited by vaginal distension has previously been demonstrated in adult mice following neonatal vaginal irritation (zymosan) and in rat and mouse models of endometriosis (Nagabukuro and Berkley, 2007; Dmitrieva et al., 2012; Castro et al., 2021). The relatively small animal-to-animal variation shown in this study supports previous assertions VMR is a highly reproducible and reliable measure of visceral nociception (Ness and Gebhart, 1988).

In summary, we have established a robust model that recapitulates key features of clinical vestibulodynia including vaginal hyperinnervation associated with an increase in the nociceptive signalling sent from the distal vagina to the CNS, and vulvovaginal hypersensitivity. In this model, clodronate administration was associated with prevention of vestibular hypersensitivity. Interventions targeting signalling pathways involving macrophages and other signalling impacted by clodronate warrant further investigation to aid understanding of chronic pain associated with vestibulodynia.

## Data Availability Statement

The original contributions presented in the study are included in the article/supplementary material. Further inquiries can be directed to the corresponding author.

## Ethics Statement

The animal study was reviewed and approved by Animal Welfare Committee of Flinders University.

## Author Contributions

CB, JC, and AH designed the project, undertook experiments, analysed data, and wrote the manuscript. FC contributed to the experiments, data collection, and analysis. All authors contributed to the manuscript design and critical revision. All authors read and approved the submitted version.

## Funding

This project was made possible by a Project Grant from the Rebecca L Cooper Medical Research Foundation (PG2019395). JC is funded by a National Health and Medical Research Council (NHMRC) of Australia Ideas Grant (APP1181448). AH received funding through the Australian Research Council (ARC) Discovery Early Career Research Award (DE130100223). SB is a National Health and Medical Research Council of Australia (NHMRC) R.D Wright Biomedical Research Fellow (APP1126378). NJS acknowledges support from NHMRC grant: 1156427

## Conflict of Interest

The authors declare that the research was conducted in the absence of any commercial or financial relationships that could be construed as a potential conflict of interest.

## Publisher’s Note

All claims expressed in this article are solely those of the authors and do not necessarily represent those of their affiliated organizations, or those of the publisher, the editors and the reviewers. Any product that may be evaluated in this article, or claim that may be made by its manufacturer, is not guaranteed or endorsed by the publisher.
